# Relationship Between Debt and Depression, Anxiety, Stress, or Suicide Ideation in Asia: A Systematic Review

**DOI:** 10.3389/fpsyg.2020.01336

**Published:** 2020-07-10

**Authors:** Noh Amit, Rozmi Ismail, Abdul Rahim Zumrah, Mohd Azmir Mohd Nizah, Tengku Elmi Azlina Tengku Muda, Edbert Chia Tat Meng, Norhayati Ibrahim, Normah Che Din

**Affiliations:** ^1^Clinical Psychology and Behavioural Health Program, Faculty of Health Sciences, Universiti Kebangsaan Malaysia, Kuala Lumpur, Malaysia; ^2^Psychology and Human Wellbeing Research Centre (PsiTra), Faculty of Social Sciences and Humanities, Universiti Kebangsaan Malaysia, Bangi, Malaysia; ^3^Faculty of Leadership and Management, Universiti Sains Islam Malaysia, Nilai, Malaysia; ^4^Pusat Permata Pintar Negara, Universiti Kebangsaan Malaysia, Bangi, Malaysia

**Keywords:** indebtedness, depression, anxiety, stress, suicide, systematic review, Asia, relationship

## Abstract

**Background:** This article aims to review research manuscripts in the past 5 years that focus on the effects of debt on depression, anxiety, stress, or suicide ideation in Asian countries.

**Methods:** A search for literature based on the PRISMA guidelines was conducted on Medline, PubMed, Web of Science, Scopus, and ScienceDirect, resulting in nine manuscripts meeting inclusion criteria. The studies were conducted in Thailand, Korea, Singapore, Pakistan, India, Cambodia, and China.

**Results:** The findings of the studies show that there is evidence to support that being in debt is related to Asian participants experiencing depression, anxiety, stress, or suicide ideation. However, the studies are limited to quantitative studies only. The definition of debt is also unclear in most manuscripts. Few manuscripts also examined how other factors influence the relationship between debt and mental illness.

**Conclusion:** There are limited studies on the psychological effects of debt on the Asian population. Future studies should focus on the relationship between debt and psychological well-being among this population.

## Introduction

The term debt can be understood in two different but related concepts, one as a specific legal instrument that connects lenders to debtors, and the other as having less assets to liabilities (Charron-Chénier and Seamster, 2018). The prevalence of debt is widespread among the youths and those of low-income groups. In Britain, younger individuals are more likely than older individuals to live in a household with debt with 60–70% of those aged 20–30 living in a household with debt compared to only 39% of those aged 60–64 and only 11% for those aged 80 to 84 (Hood et al., 2018). As income increases, so does the prevalence of debt; research shows among among individuals in the lowest income decile, 7% live in households with debts higher than 10,000 euros, and this value increases to 18% among those of the highest income decile, where more than 60% of unsecured debt are held by above-average incomes (Hood et al., 2018). Some studies that relate age to debt found that credit card debt peaks among individuals aged 40–49 (Drentea, 2000). A more recent finding indicates that among individuals aged 18–30, the amount of debt increases with age (Hoeve et al., 2014). In an Asian context, studies from Malaysia find individuals aged below 40 make up the largest number of borrowers with debt at risk (Siti et al., 2018) while most professionals aged 30 to 40 are faced with credit card defaults (Ahmad and Omar, 2013). The Malaysian Department of Insolvency in 2016 reported a total of 101,958 cases of bankruptcy from 2012 to 2016, with individuals aged 25–34 making up 23.38% of the total bankruptcy cases (Malaysian Department of Insolvency, 2019). There is a need to understand the effects of debt on humans as research finds that individuals can be affected physically and mentally by their debts.

Arandjelovic et al. (2016) drew attention to the disparity in mental health care in Asia compared to Australia, and even calls for Australia's involvement in improving the mental health outcomes in the Asian region. World Health Organization (2018) estimates that South-East Asia has the second highest suicide rate compared to other regions. Evidence of this prevailing issue can be seen in Malaysia, whereby 31.3% of adolescents have suicide ideation (Ibrahim et al., 2019) which correlates positively with depression, anxiety, and stress (Ibrahim et al., 2014). Hence, a deeper understanding of the factors that contribute to these mental health issues is needed, especially debt.

In terms of depression, the amount of debt is not the sole predictor of depression. It is found that among the older adults in Japan, having debt was significantly related to the increase in mild–moderate and severe depression; this is attributed to the obligation to repay debt results in psychological pain or reduced quality of living conditions (Tatsuhiko et al., 2010). Bridges and Disney (2010) found that person-specific effects are important in the relationship between self-reported debts and depression. Hojman et al. (2016) reported that over-indebtedness is positively associated with depression; more specifically, the duration of over-indebtedness predicts depressive symptoms.

In studies of anxiety, Dackehag et al. (2019) reported that debt was significantly associated with anxiety. Additionally, among indebted Union Cross members in low-income households, the rate of anxiety was higher compared to the Northern Ireland average, with 11.5% of the participants describing themselves as anxious or depressed, and 23.5% receiving treatment for anxiety or depression (Keatley, 2014). Qualitatively, a study using 14 focus groups of low- to middle-income house threatened with foreclosure and foreclosure intervention professionals reports changes in mental health as a result of stress and anxiety due to their financial hardship, their efforts to manage the financial problem, and loss of ontological support (Libman et al., 2012).

Additionally, there have been numerous past studies on the effect of debt on stress. Norvilitis et al. (2006) reported that debt (in the form of credit card and store debt) among individuals aged 18 and above and college students predicted overall stress. Head of homes with outstanding credit either at the household or individual level also report significant lower psychological well-being, and the presence of household debt lowers the chances of scoring full marks on the General Health Questionnaire 12 by 6% (Brown et al., 2005). In a diverse sample of Internet users, it was found that those that fall in the foreclosure or default group experienced greater psychological distress than other groups (Cannuscio et al., 2012).

In terms of suicide behavior, although not observed as a statistically significant pattern, debt was mentioned as a relevant factor for suicide behavior in 11% of men by inquest witnesses (Scourfield et al., 2012). In Hong Kong, a study of suicide by gassing from 2005 to 2013 reported that those that employed suicide by helium were more likely to have debt, and debt was associated with charcoal suicide as well (Chang et al., 2016). Hopelessness acts as a partial mediator between debt and suicide and ideation. However, Kidger et al. (2011) noted that suicide attempts were more strongly associated with bankruptcy within 2 years compared to preinjury bankruptcy; nonetheless, the weaker association is still significant for preinjury bankruptcy and suicide.

Higher debt/income ratio is significantly related to worsening health and self-reported health with health behaviors and risk explaining part of the association between debt, stress about debt, and health (Drentea and Lavrakas, 2000). According to Angel (2016), among several European countries, those living in households with debt for the past 12 months significantly increase the chances of reporting bad health by 22.6% compared to those living without debt, and there is evidence that debt collection costs strengthen the relationship between debt and health, while the evidence of the effect of social stigma is weak.

From these past studies, there is evidence to support a need for a deeper understanding of how debt affects the mental health of individuals. One such method is through a systematic review, a form of review that involves a thorough and comprehensive plan using a search strategy with the aim of lowering bias by locating, evaluating, and synthesizing all relevant studies on the studied topic (Uman, 2011). There have been systematic reviews on the effects of debt. One is Richardson et al. (2013) meta-analysis of research manuscripts on the effects of debt, which found that there is a significant relationship between the presence of mental disorders, suicide attempts and completion, depression, neurotic disorders, psychotic disorders, drug dependence, and problematic alcohol use. In their study, however, only 10 manuscripts were found that were conducted in countries in Asia. Turunen and Hiilamo (2014) also reviewed the effects of debt and concluded that indebted individuals suffered from suicide ideation and depression more than those without debt; in their research, the authors did not include any manuscripts conducted in the Asian region.

Based on previous literature, it can be derived that debt plays an important part in the mental and physical health of humans. However, there are conceptual and cultural issues to be addressed in reviewing literature on debt and mental health across cultures. First, in terms of conceptual definitions of debt and measurements of debt—the use of clear definition of debts and measures of multidimensional domains of debt may facilitate the accuracy in measuring debt or loan. The use of multidimensional domains of debt are better compared to the use of single-item response to measure debt (Roth et al., 2008). The comprehensive measure of debt potentially leads to meaningful data especially in understanding the relationship between debts and mental health. Second, cultural roles are important in explaining the relationship between debts and mental health in societies and mental health differences across cultures. Therefore, examining ethnicity, culture, and cultural background in a systematic review may offer a cultural explanation on the relationship between debts and mental health (Gopalkrishnan and Babacan, 2015). To our knowledge, few studies on the effects of debt have been done in Asia. It would add to the body of knowledge to see if these patterns of psychological effects on indebted individuals apply for Asian participants. Conducting a review on the effects of debt specifically in Asia will enhance knowledge about how research into debt has changed among Asian countries, and if there is potential to enhance the research methodology. Therefore, this review aims to investigate the relationship between debt, depression, anxiety, stress, and suicide ideation in Asia.

## Methods

### Systematic Review Protocol

This review follows the PRISMA guidelines, and the PRSIMA flowchart was also adapted to summarize the search process (Moher et al., 2009). PRISMA is the revised version of the Quality of Reporting of Meta-analyses (QUAROM) guideline, consisting of a 27-item checklist and flowchart (Moher et al., 2009). PRISMA's strong endorsement has resulted in an increase in adherence in PRISMA's guidelines within systematic reviews and meta-analysis, and it also found an increase in qualities in manuscripts that endorse PRISMA regardless of their declaration of adopting its methods or not (Panic et al., 2013).

### Search Strategy

Five databases were searched. These are Medline, PubMed, Web of Science, Scopus, and ScienceDirect. Generally, a systematic review requires the use of more than two databases and should go beyond the use of MEDLINE database (Charrois, 2015). In Bramer et al. (2017) manuscript, Medline appears in 97% of systematic reviews identified via PubMed. Medline contains more than 25 million references on the topics related to biomedicine and life science (U.S. National Library of Medicine, 2019a). Similarly, PubMed archives biomedical and life science journals literature and is available as a free resource (U.S. National Library of Medicine, 2019b). Web of Science covers numerous records on social, biomedical, life science, natural sciences, engineering, computer science, material sciences, and health sciences (Web of Science Group, 2019). Scopus is a free-to-use database of peer-reviewed literature with content in the field of science, social science, technology, medicine, and arts and humanities (Elsevier, 2017). ScienceDirect provides access to journals and books on the field of social sciences, business, health sciences, life sciences, physical sciences, and engineering (Harnegie, 2013).

The search terms used were debt^*^ or indebtedness or over-indebtedness or credit or loan or “financial problems” or bankrupt and Asia and “mental disorder” or “mental health” or “mental illness” or depression or anxiety or stress or suicide or “suicide ideation.”

The EBSCOhost search engine was used for the MEDLINE database search, and the following limiters were used as they were ready-made and to enhance accuracy of search results: Age set to 19–44 years old, and geography set to India, China, Malaysia, Japan, Thailand, Bangladesh, and Republic of Korea.

Third, the published journal article discusses the relationship between problematic and non-problematic debt on depression, anxiety, suicide or suicide ideation, or stress. The study includes studies on participants from Asia only.

The following exclusion criteria were followed. First, the manuscript was excluded if the content was not available in English. This limiter is considered acceptable as research finds no bias in systematic reviews of meta-analysis of conventional medicine that apply the language restriction (Morrison et al., 2012). Second, if the manuscript's study was not conducted in a country in Asia. Third, the manuscript was a review manuscript. The reasoning to reject review manuscripts is because as a form of secondary research, the use of secondary data would limit the quality of the data as the data originally would have been used for other purposes that may not be aligned with the current research (Allen, 2017). Hence, the present review chooses to focus on primary research data.

It was decided that the review would focus on published journal articles, as it was judged to be biased if books and thesis or dissertations on the subject matter were selected over the other due to their unavailability via online search and written language.

### Data Analysis

Each accepted manuscript for review was analyzed through a systematic and careful process. The full text of the articles was read, exploring their methodology and results. Information on the study's design, sampling method, sample size, psychological tools used, and definition of debt was recorded. Additionally, results on the relationship between debt and depression, anxiety, suicide, and suicide ideation were noted. All relevant findings are categorized and presented in a descriptive method in Tables 1–3. A risk of bias analysis was carried out for each individual study as well and summary of risk of bias analysis is presented in Table 4. The summary of the PRISMA checklist and a sample for electronic search strategy for the present systematic review are presented in Tables 5, 6, respectively.

**Table 1:** Result Table.

**References**	**Design**	**Sample selection method**	**Sample size**	**Tool(s)**	**How debt is measured**	**Results**
Kaufman (2015)	Quantitative cross sectional	Purposive sampling method	*N* = 139 farmers of Ubon Ratchathani Province (75 organic and 64 non-organic)	1. Questionnaire developed from interview results conducted by researcher in a prior research and the National Institute of Health (North America). 2. Information gathered on participants' households, environmental views. Perception of well-being, production methods.	Debt is measured by the severity level of loans the participant has.	Non-organic farmers in Ubon Ratchathani were significantly happier than organic farmers. However, both organic and non-organic farmers experience depression, or sadness in relation to their debts.
Lee et al. (2016)	Quantitative, longitudinal study	Stratified sampling	*N* = 7565 individuals from Korea age 19 and above	1. The Center for Epidemiologic Studies Depression Scale (CESD-11) 2. Three questions measure house poor	Debt was defined by ratio of interest paid in household debts to disposable income.	After adjusting for covariates, the higher the house-related interest to disposable income ratio people with houses had, the higher the depression scores. In the middle-low equalized income group, people with over 10% house-related interest to disposable income ratios had significantly higher depression scores than people without houses when setting people with houses and no debts as the reference. In the low-income group, regardless of house possession or related interest status, people had noticeably higher depressive symptoms than individuals in other income groups except for people in the under 5% house related interest group
Manning et al. (2015)	Quantitative cross sectional	Purposive sampling	*N* = 2187 participants of a Treatment Outcome Monitoring program as part of the National Addictions Management Service for substance or Behavioral addiction problems. Diagnosed with primary alcohol use disorder, drug use disorder, or gambling disorder or problematic gambling in Singapore.	1. The Addiction Severity Index-Lite (ASI-Lite) 2. The Gambling Symptom Assessment Scale (G-SAS) 3. Personal Wellbeing Index 4. Questions about suicidal intent, plans, and attempts.	Debt was not defined.	Participants with debt were 1.9 times more likely to report suicidal thoughts in the past month, 1.6 times more likely to report a suicidal plan, and were 1.6 times more likely to attempted suicide.
Maselko et al. (2018)	Quantitative, cross sectional.	Cluster randomization	*N* = 1154 women in their third trimester of pregnancy at baseline who were randomly selected for a perinatal depression intervention in Pakistan average age of 26.6 (18–45)	1. Patient Health Questionnaire (PHQ-9)	Debt was measured as a Yes/No/Unknown question whether the household was in debt.	Being in a family in debt was associated with a 2.08-point higher PHQ-9 score. Debt continued to independently predict depression symptoms together with the asset index in the current study. There was weak evidence that the association between debt and depression symptoms was stronger among those toward the bottom of the asset score distribution, although this difference did not reach statistical significance (results not shown).
Mathias et al. (2015)	Quantitative cross sectional	Randomized cluster sampling.	*N* = 958 individuals from North India with mean age of 39.4 (18–60)	1. Patient Health Questionnaire (PHQ9) 2. Questions about General health help-seeking behavior and health service utilization 3. Questions about talking therapy or medication prescription received 4. Adapted questions from the Client Service Receipt Inventory	Debt is not well defined, but is implied is the loans participants take in the last 6 months.	Depression prevalence among people who had taken a recent loan was thrice that of those who had not.
Seponski et al. (2018)	Quantitative cross sectional	Multi stage Cluster sampling	*N* = 2690 Cambodians age 21 and up.	1. Harvard Trauma Questionnaire (HTQ) 2. Hopkins Symptom Checklist 25	Debt is not defined.	Respondents who were in debt had the highest percentage of anxiety (35.26%). Similarly, respondents who were in debt had the highest percentage of depression (27.75%) significant at (*p* <0.05)
Sharma et al. (2018)	Quantitative cross sectional	Purposive sampling	*N* = 100, 50 caregivers of Schizophrenia and 50 Bipolar Affective Disorder patients	1. Modified Caregiver Stress Index (MCSI) 2. Nepali version of Beck‘s Depression Inventory (BDI) 3. Beck‘s Anxiety Inventory (BAI)	How debt is measured is not defined. However, this research refers to debt due to illness.	Caregiver strain was found to be high and statistically significant with being in debt.
Shidhaye et al. (2016)	Quantitative cross sectional	Systematic random sampling	*N* = 1456 individuals from Chandur Bazaar and Shamangaon taluka age 18-87.	1. Marathi version of Patient Health Questionnaire (PHQ-9). 2. Questions on suicidal ideation which was adapted from the Mini International Neuropsychiatric Interview (MINI)	No definition of debt but implied it refers to loan.	The risk of depression was one and half times in individuals belonging to households below poverty line and it was double in those who were in debt.
Xu et al. (2015)	Quantitative, cross sectional	Cluster sampling	*N* = 4291 Individuals from China age 20-65 and above.	1. Question about suicidal ideation (Yes/No) in the past 12 months. 2. 20-item Center for Epidemiologic Studies Depression Scale (CES-D) Chinese version	Debt is measured as a Yes/No question.	Being in debt is significantly positively associated with suicidal ideation, for women being in debt is related to suicide ideation within 12 months.

**Table 2:** The characteristics and culture background of participants.

**Study**	**Country**	**Locality of sample**	**Sample income status**	**Sample education status**	**Sample ethnic group distribution**
Kaufman (2015)	Thailand	Rural	22.7% earning 0–40,000 Baht.	50.7% with 6th grade or less education.	Distribution by ethnic group is not reported.
			12.0% earning 41,000–60,000 Baht.	49.3% with 7th grade or more education.	
			28.0% earning 61,000– 100,000 Baht.		
			21.3% earning 101,000–200,000 Baht.		
			16.0% earning 201,000 or more.		
Lee et al. (2016)	Korea	Rural and non-rural	24.1% households with low income.	41.3% with less than high school education.	Distribution by ethnic group is not reported.
			24.3% households with middle low income	30.8% with high school graduate education.	
			25.6% households with middle high income.	27.8% with college graduate education.	
			26.0% households with high income.		
Manning et al. (2015)	Singapore	Not reported	Participants' income is not reported.	68.7% educated to secondary school or above.	61.3% Chinese
					9.7% Indian
					5.6% Malay
					3.4% are of other races.
Maselko et al. (2018)	Pakistan	Rural	Participant's income is not reported. Study uses an asset index score as a measure of socioeconomic status.	18.8% women with no education.	Distribution by ethnic group is not reported.
				24.4% women with primary education.	
				18.8% women with middle education.	
				22.1% women with higher secondary education.	
				8.1% women with higher secondary education.	
				7.9% with tertiary education.	
				9.6% men with no education.	
				11.8% men with primary education.	
				24.0% men with middle education.	
				42.6% men with secondary education.	
				8.2% with higher secondary education.	
				3.7% with tertiary education.	
Mathias et al. (2015)	India	Rural and non-rural	Participant's income is not reported.	22.7% with none or incomplete primary education.	25.4% Scheduled Caste/Tribe
				18.3% with primary completion.	15.4% Other Backward Caste
				41.5% with secondary completion.	59.2% General
				17.5% with graduate education.	
Seponski et al. (2018)	Cambodia	Rural and non-rural	Participant's income is not reported.	18.1% no education	Distribution by ethnic group is not reported.
				47.6% primary school	
				21.7% secondary school	
				12.6% high school and beyond.	
Sharma et al. (2018)	Nepal	Not reported	Participant's income is not reported.	36% caregivers of schizophrenia patients with education up to grade 12.	Distribution by ethnic group is not reported.
				64% caregivers of schizophrenia patients who graduated and above.	
				24% caregivers of bipolar affective disorder patients with education up to grade 12.	
				76% caregivers of bipolar affective disorder patients who graduated and above.	
Shidhaye et al. (2016)	India	Rural	In Dhamangaon	In Dhamangaon	In Dhamangaon
			12.0% first quintile annual income.	6.5% graduated and above.	56.9% other backwards caste.
			18.4% second quintile annual income.	17.0% junior college.	15.4% schedule caste.
			13.7% third quintile annual income.	29.5% high school.	11.3% schedule tribe.
				31.9% primary and middle school.	16.3% general.
				15.1% illiterate.	In Chandur Bazaar
					52.9% other backwards caste.
					23.1% schedule caste.
				In Chandur Bazaar	4.5% schedule tribe.
				9.2% graduated and above.	19.4% general.
			30.1% fourth quintile annual income.	12.0% junior college.	
				29.3% high school.	
			25.7% fifth quintile annual income.	36.1% primary and middle school.	
			In Chandur Bazaar	13.4% illiterate.	
			27.2% first quintile annual income.		
			27.2% second quintile annual income.		
			24.3% third quintile annual income.		
			14.0% fourth quintile annual income.		
			7.3% fifth quintile annual income.		
Xu et al. (2015)	China	Non-rural	Participants' income not reported.	Non-suicide ideation 96.25% junior high or less 96.32% high school 97.02% college or above With suicidal ideation 4.75% junior high or less 3.68% high school 2.98% college or above	Distribution by ethnic group is not reported.

**Table 3:** The types, definitions and measurement of debts.

**Study**	**Was the type of debt clearly stated?**	**Debt definition**	**Debt measurement technique**
Kaufman (2015)	Yes	Debt was defined measured as loan status over five years.	Participants self-report on a Likert scale, the higher the value the lower the loan.
Lee et al. (2016)	Yes	Debt was defined by ratio of interest paid in household debts to disposable income.	Participants answer three questions. First, what was their house ownership category? Second, at the end of last year how much house related debt interest was paid. Third, inquired on the household income.
Manning et al. (2015)	No	Debt was not defined.	Debt measuring method was not reported. However, data reported on debt is presented as a Yes or No response.
Maselko et al. (2018)	No	Debt was not defined.	Participants self-report on a single question on whether the household was in debt. Participants responses fell into categories of Yes, No or Unknown.
Mathias et al. (2015)	No	Debt was defined as loans in the last 6 months.	Participants self-report in a survey whether Yes or No they had loans in the last 6 months.
Seponski et al. (2018)	No	Debt was not defined.	Participants self-reported on a structured interview on their monthly saving per capita, participants responses fell into three categories; In debt, savings, or in debt and no savings.
Sharma et al. (2018)	Yes	Debt was referred as debt due to illness.	Participants self-reported in an interview whether they had taken or not taken debt due to illness.
Shidhaye et al. (2016)	No	Debt was not defined.	Participants self-reported Yes or No if they had loans.
Xu et al. (2015)	No	Debt was not defined.	Participants self-reported Yes or No if their family had any debts in the past year

**Table 4:** Summary of Risk of Bias.

**Criteria**	**Kaufman (2015)**	**Lee et al. (2016)**	**Manning et al. (2015)**	**Maselko et al. (2018)**	**Mathias et al. (2015)**	**Seponski et al. (2018)**	**Sharma et al. (2018)**	**Shidhaye et al. (2016)**	**Xu et al. (2015)**
1. Was the research question or objective in this paper clearly stated?	Yes	Yes	Yes	Yes	Yes	Yes	Yes	Yes	Yes
2. Was the study population clearly specified and defined?	Yes	Yes	Yes	Yes	Yes	Yes	Yes	Yes	Yes
3. Was the participation rate of eligible persons at least 50%?	Yes	NR^*^	Yes	NR^*^	No^*^	NR^*^	NR^*^	NR^*^	NR^*^
4. Were all the subjects selected or recruited from the same or similar populations (including the same time period)? Were inclusion and exclusion criteria for being in the study prespecified and applied uniformly to all participants?	Yes	Yes	Yes	Yes	Yes	Yes	Yes	Yes	Yes
5. Was a sample size justification, power description, or variance and effect estimates provided?	No	No	No	No	Yes	Yes	No	Yes	No
6. For the analyses in this paper, were the exposure(s) of interest measured prior to the outcome(s) being measured?	No	Yes	Yes	No	No	No	No	No	No
7. Was the timeframe sufficient so that one could reasonably expect to see an association between exposure and outcome if it existed?	No	Yes	No	No	No	No	No	No	No
8. For exposures that can vary in amount or level, did the study examine different levels of the exposure as related to the outcome (e.g., categories of exposure, or exposure measured as continuous variable)?	NA^*^	Yes	NA^*^	Yes	Yes	Yes	NA^*^	Yes	Yes
9. Were the exposure measures (independent variables) clearly defined, valid, reliable, and implemented consistently across all study participants?	No	Yes	Yes	Yes	Yes	Yes	Yes	Yes	Yes
10. Was the exposure(s) assessed more than once over time?	NA^*^	Yes	NA^*^	NA^*^	NA^*^	NA^*^	NA^*^	NA^*^	NA^*^
11. Were the outcome measures (dependent variables) clearly defined, valid, reliable, and implemented consistently across all study participants?	Yes	Yes	No	Yes	Yes	Yes	Yes	Yes	Yes
12. Were the outcome assessors blinded to the exposure status of participants?	No	NR^*^	No	No	No	No	No	NR^*^	No
13. Was loss to follow-up after baseline 20% or less?	NA^*^	No	NA^*^	NA^*^	NA^*^	NA^*^	NA^*^	NA^*^	NA^*^
14. Were key potential confounding variables measured and adjusted statistically for their impact on the relationship between exposure(s) and outcome(s)?	No	Yes	Yes	Yes	Yes	Yes	Yes	Yes	Yes
Overall evaluation	Fair	Good	Good	Good	Good	Good	Good	Good	Good

NA^*^, Not applicable; NR^*^, Not reported; No^*^, Does not fulfil criteria but justified with reasons.

**Table 5:** PRISMA Checklist.

**Section/topic**	**No**	**Checklist item**	**Reported on page No**
**TITLE**			
Title	1	Identify the report as a systematic review, meta-analysis, or both.	Title
**ABSTRACT**			
Structured summary	2	Provide a structured summary including, as applicable: background; objectives; data sources; study eligibility criteria, participants, and interventions; study appraisal and synthesis methods; results; limitations; conclusions and implications of key findings; systematic review registration number.	Abstract
**INTRODUCTION**			
Rationale	3	Describe the rationale for the review in the context of what is already known.	Introduction
Objectives	4	Provide an explicit statement of questions being addressed with reference to participants, interventions, comparisons, outcomes, and study design (PICOS).	Introduction
**METHODS**			
Protocol and registration	5	Indicate if a review protocol exists, if and where it can be accessed (e.g., Web address), and, if available, provide registration information including registration number.	Methods
Eligibility criteria	6	Specify study characteristics (e.g., PICOS, length of follow-up) and report characteristics (e.g., years considered, language, publication status) used as criteria for eligibility, giving rationale.	Methods
Information sources	7	Describe all information sources (e.g., databases with dates of coverage, contact with study authors to identify additional studies) in the search and date last searched.	Methods
Search	8	Present full electronic search strategy for at least one database, including any limits used, such that it could be repeated.	Methods
Study selection	9	State the process for selecting studies (i.e., screening, eligibility, included in systematic review, and, if applicable, included in the meta-analysis).	Methods
Data collection process	10	Describe method of data extraction from reports (e.g., piloted forms, independently, in duplicate) and any processes for obtaining and confirming data from investigators.	Methods
Data items	11	List and define all variables for which data were sought (e.g., PICOS, funding sources) and any assumptions and simplifications made.	Methods
Risk of bias in individual studies	12	Describe methods used for assessing risk of bias of individual studies (including specification of whether this was done at the study or outcome level), and how this information is to be used in any data synthesis.	Methods
Summary measures	13	State the principal summary measures (e.g., risk ratio, difference in means).	–
Synthesis of results	14	Describe the methods of handling data and combining results of studies, if done, including measures of consistency (e.g., I2) for each meta-analysis.	–
Risk of bias across studies	15	Specify any assessment of risk of bias that may affect the cumulative evidence (e.g., publication bias, selective reporting within studies).	–
Additional analyses	16	Describe methods of additional analyses (e.g., sensitivity or subgroup analyses, meta-regression), if done, indicating which were pre-specified.	–
**RESULTS**			
Study selection	17	Give numbers of studies screened, assessed for eligibility, and included in the review, with reasons for exclusions at each stage, ideally with a flow diagram.	Results
Study characteristics	18	For each study, present characteristics for which data were extracted (e.g., study size, PICOS, follow-up period) and provide the citations.	Results
Risk of bias within studies	19	Present data on risk of bias of each study and, if available, any outcome level assessment (see item 12).	Results
Results of individual studies	20	For all outcomes considered (benefits or harms), present, for each study: (a) simple summary data for each intervention group (b) effect estimates and confidence intervals, ideally with a forest plot.	Supplementary material
Synthesis of results	21	Present results of each meta-analysis done, including confidence intervals and measures of consistency.	Results
Risk of bias across studies	22	Present results of any assessment of risk of bias across studies (see Item 15).	Results
Additional analysis	23	Give results of additional analyses, if done [e.g., sensitivity or subgroup analyses, meta-regression (see Item 16)].	–
**DISCUSSION**			
Summary of evidence	24	Summarize the main findings including the strength of evidence for each main outcome; consider their relevance to key groups (e.g., healthcare providers, users, and policy makers).	Discussion
Limitations	25	Discuss limitations at study and outcome level (e.g., risk of bias), and at review-level (e.g., incomplete retrieval of identified research, reporting bias).	Discussion
Conclusions	26	Provide a general interpretation of the results in the context of other evidence, and implications for future research.	Discussion
**FUNDING**			
Funding	27	Describe sources of funding for the systematic review and other support (e.g., supply of data); role of funders for the systematic review.	Funding

**Table 6:** PubMed Search Strategy.

**Database searched**	**Date of search**	**Search terms**	**Filters/Limiters applied**	**No. of records retrieved**	**No. of records excluded after screening**	**No. of records included**
PubMed	16 October 2019	(debt OR indebtedness OR over-indebtedness OR credit OR loan OR “financial problems” OR bankrupt) AND Asia AND (“mental disorder” OR “mental health” OR “mental illness” OR depression OR anxiety OR stress OR suicide OR “suicide ideation”)	Years: 2015–2019	56	48	8

## Results

### Flow Diagram

**Figure 1:**
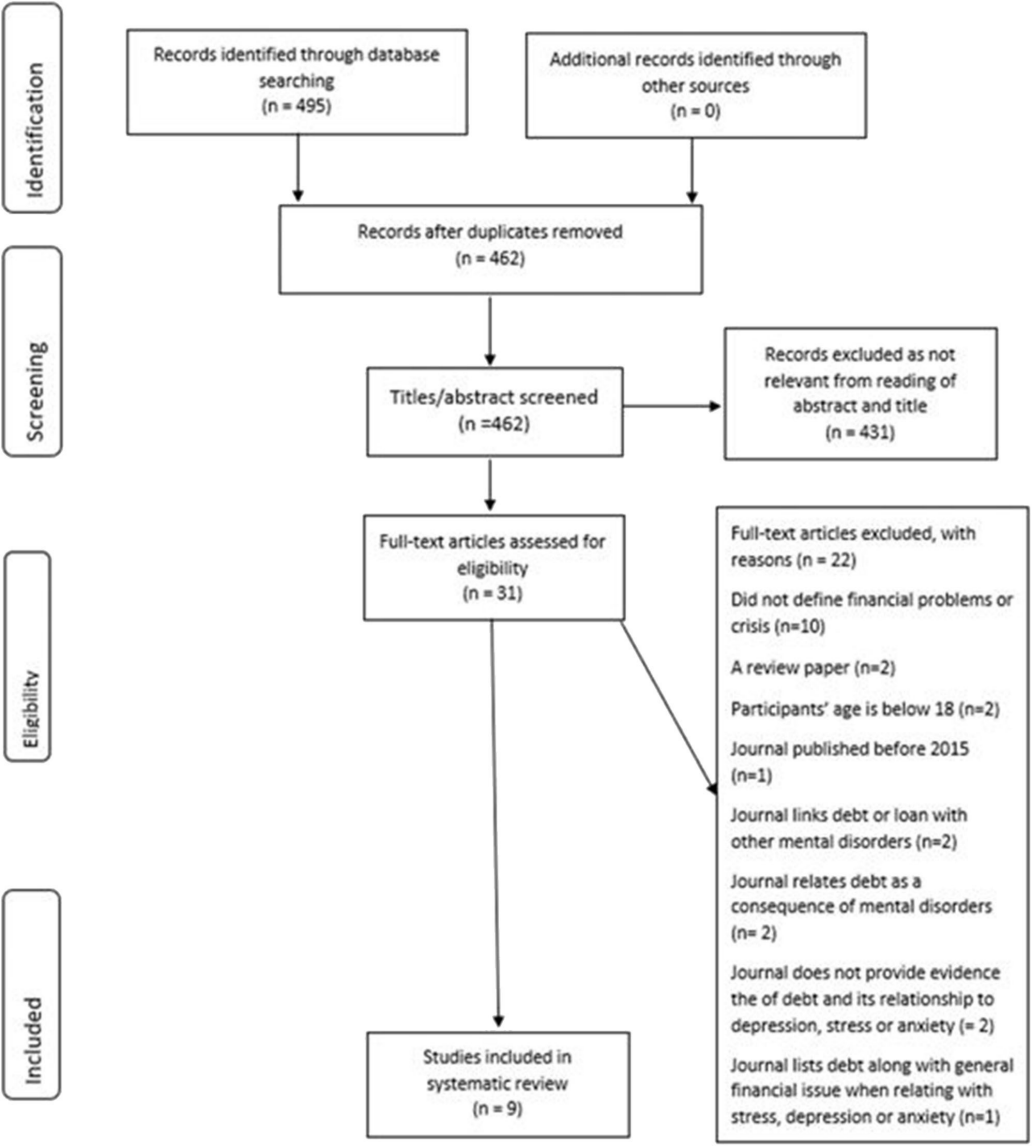
PRISMA flow chart.

See Figure 1 for the flow diagram of study selection.

### Study Selection and Characteristics

The identification search process was conducted from October 9, 2019 to October 10, 2019 for the Medline, PubMed, Scopus, and Web of Science database. ScienceDirect was searched on October 25, 2019. Using the search terms, a total of 462 articles were found from all five databases after excluding duplicates. During the first screening process, the title and abstract of each manuscript were screened for relevance and 431 titles were omitted.

Studies that were deemed acceptable were then screened for eligibility via the full text for their methodology and their findings. From the reading of the full text, several manuscripts were excluded with various reasons. These included manuscripts that discussed the effects of debt on other mental illnesses such as gambling addiction and post-traumatic embitterment, manuscripts that relate debt as a consequence of mental illnesses, manuscripts that mention a relationship between debt and depression, anxiety, suicide, or suicide ideation via citing other sources or presenting sample characteristics alone, manuscripts with participants below the age of 18, manuscripts that do not define the term financial problems or crisis, manuscripts that are review manuscripts as these are secondary sources, and manuscripts that combine debt with other financial problems to relate with mental disorders.

The number of manuscripts included and deemed suitable for review was nine. See Figure 1 for the PRISMA flow diagram.

### Synthesized Findings

A total of nine studies were selected for review. All studies (100%) utilized a qualitative design, with only 11.11% using longitudinal data while the remaining 88.88% used cross-sectional data.

Overall, the most studied relationship is between debt and depression, with six out of nine manuscripts measuring depression as part of their study, accounting for 66.67% of the total manuscripts (Kaufman, 2015; Mathias et al., 2015; Lee et al., 2016; Shidhaye et al., 2016; Maselko et al., 2018; Seponski et al., 2018).

Four out of nine or 44.44% explored the relationship between debt and depression alone. One study or 11.11% of the found manuscripts examined the relationship between debt depression, anger, and sadness. The found manuscripts reported that those in debt experience depression, and there is evidence that those in debt experience greater debt than those without.

Only one study, or 11.11% of the found manuscripts explored the relationship between debt and depression and anxiety. Seponski et al. (2018) reported that participants who reported being in debt had the highest level of anxiety.

Two manuscripts or 22.22% of the manuscripts studied the relationship between debt and suicide ideations and behavior. Manning et al. (2015) found that patients with addictive disorders were almost twice as likely to have suicidal thoughts and were 1.6 times more likely to have suicidal plans or attempted suicide. Xu et al. (2015) found that being in debt was positively related to suicide ideation.

Lastly, only one manuscript or 11.11% of the nine manuscripts discussed the relationship between debt and stress. The participants of this study are highly specific and limited to caregivers of inpatients in a hospital, and the manuscript's findings found that being in debt was statistically significant with caregiver stress.

In addition, this review examines the characteristics and culture background of participants reported by the studies. Table 2 indicates that, in terms of the geographic location of the studies, 33.33% were conducted with participants from rural locations (Kaufman, 2015; Shidhaye et al., 2016; Maselko et al., 2018), 33.33% were conducted with participants from rural and non-rural locations (Mathias et al., 2015; Lee et al., 2016; Seponski et al., 2018), 11.11% were conducted with participants from non-rural locations only (Xu et al., 2015), and 22.22% did not report the geographical location the participant was recruited from (Manning et al., 2015; Sharma et al., 2018). In terms of income status, only 33.33% of the found manuscripts provide information on the participant's income status and majority of the participants are of middle- to low-income groups (Kaufman, 2015; Lee et al., 2016; Shidhaye et al., 2016). In terms of education status, 100% of the manuscripts provided information on the participants' education status (Kaufman, 2015; Manning et al., 2015; Mathias et al., 2015; Xu et al., 2015; Lee et al., 2016; Shidhaye et al., 2016; Maselko et al., 2018; Seponski et al., 2018; Sharma et al., 2018). Majority of the participants are of low education status. With regard to ethnic group distribution, only 33.33% of the manuscripts reported on the samples ethnic group distribution (Manning et al., 2015; Mathias et al., 2015; Shidhaye et al., 2016). This may limit the understanding of the cultural background of study participants in the present review and potentially limits knowledge on cultural explanation on the relationship between debts and mental health.

Besides that, this present review identifies types of debts, definition of debts, and methods of measuring debt in order to understand the variability in defining and measuring debts as indicated in Table 3. First, with regard to reporting the type of debt, only 33.33% of studies provide a clear statement of what type of debt is referred to in the study (Kaufman, 2015; Lee et al., 2016; Sharma et al., 2018), while 66.66% of studies do not state what type of debt they refer to or in the writing require readers to infer the type of debt from later sections of the manuscript (Manning et al., 2015; Mathias et al., 2015; Xu et al., 2015; Shidhaye et al., 2016; Maselko et al., 2018; Seponski et al., 2018). Second, in terms of definitions of debts, only 44.44% of studies provide a definition of the type of debt the study looks into (Kaufman, 2015; Mathias et al., 2015; Lee et al., 2016; Sharma et al., 2018). For instance, while Kaufman (2015) defined debt as loan status over 5 years, Lee et al. (2016) defined debt as house-related interest to disposable income ratios. The other studies defined debt as loans in the last 6 months (Mathias et al., 2015) and debt due to illness (Sharma et al., 2018). Thirdly, in terms of how debt was measured, all manuscripts used self-report measures. In addition, 55.55% of the found manuscripts obtained information on debt by asking Yes or No type questions (Manning et al., 2015; Mathias et al., 2015; Xu et al., 2015; Shidhaye et al., 2016; Maselko et al., 2018). Other studies measured debt on a Likert scale, indicating how severe the loans were (Kaufman, 2015), and one study measured savings per capita (Seponski et al., 2018); each of these studies account for 11.11% of the nine manuscripts included in this review. With regard to the method the studies used to define debt, this systematic review finds that there are variabilities in defining and measuring debts.

### Risk of Bias

Risk of bias of each individual study is determined using the National Heart, Lung, and Blood Institute (2019) Quality Assessment Tool for Observational Cohort and Cross-Sectional Studies. This guideline has been used in previous reviews (Harris et al., 2016; Koppen et al., 2016; Connolly et al., 2017; Carbia et al., 2018).

The information from this risk of bias assessment aims to evaluate the quality of the research manuscripts included in the present review; regardless of the manuscript's evaluation, its strengths and weaknesses are used to generate methods to enhance future studies and to inform on the current state of research on the psychological effects of debt.

From the nine manuscripts, a noticeable trend is seen in the reporting of sample size. Only 25% of the found studies reported how the sample size was justified while others did not; this calls into question whether the sample used is reflective of the population as only 22.22% of the studies reported the participation rate of eligible participants.

The use of cross-sectional designs also results in an increase in bias due to the nature of the design itself. One of the limitations that is seen across all the cross-sectional studies is that causation cannot be established. This is seen in 77.78% of the total found manuscripts. Due to the nature of cross-sectional analyses, these studies do not allow enough time for an independent variable to have an effect or to occur or to be observed (National Heart, Lung, and Blood Institute, 2019).

A strength of the found manuscripts is that 88.88% of the found manuscripts used reliable, valid, or objective means of measure for their independent or dependent variable in line with the objectives of their study. However, this is not reflective of how specifically debt is measured in these studies. A few manuscripts define debt clearly and use self-reports or measure debt as a dichotomous variable, which may not accurately reflect the experience of debt. The most used tool is the Patient Health Questionnaire-9 (PHQ-9) in the study of depression; the PHQ-9 has been shown to have good internal consistency and acceptable inter-item correlations, and strong convergent validity with the PHQ-2 (Maroufizadeh et al., 2019). Evidence also shows acceptable evidence of validity and reliability in translated versions of the PHQ-9 (Lupascu et al., 2019). However, from the two studies of debt and suicide ideation, one employed a Yes/No question in the measure of suicide ideation; a concern that can be raised is that the context of the suicide ideation is lost in the dichotomous choice. It can be argued that there is no gold standard for the measure of suicide ideation; however, there are numerous tools with reported validity and reliability that can be used to enhance the results of such research and should be considered in the future (Ghasemi et al., 2015).

See Table 4 for a summary of the bias table.

## Discussion

### Summary of Main Findings

This review finds that few researches on the topic of debt and depression, anxiety, stress, or suicide were carried out in Asian countries. All the reviewed manuscripts use a quantitative design and a majority use cross-sectional data. The relationship between debt and depression is studied most frequently. The majority of the manuscripts did not define debt in their manuscripts. From these findings, a few points need to be addressed in terms of the methodology and findings of these manuscripts.

First, there is a need for more research into how debt impacts the mental health of the Asian population as there are very few recent manuscripts that explore this current topic. Simultaneously, focus on the studies of the psychological impact of debt should extend beyond depression, as the current manuscript finds a majority of the studies with Asian population mostly relate debt to depression (Kaufman, 2015; Mathias et al., 2015; Lee et al., 2016; Shidhaye et al., 2016; Maselko et al., 2018; Seponski et al., 2018).

Second, there is a need for more research into the roles of culture in understanding the relationship between debt and mental health. In the present review, there was lack of evidence on understanding the cultural explanation on the relationship between debts and mental health. This review finds that more research is needed in understanding of how cultural background such as socioeconomic factors impact the psychological effects of debt among Asians. The current manuscripts show some support that being in lower socioeconomic status increases the risk of depression due to debt (Lee et al., 2016; Shidhaye et al., 2016; Maselko et al., 2018). However, the majority of the found manuscripts do not report on this association. This is cause for concern as there is evidence that those in the lower socioeconomic group are more vulnerable to incurring debt (Kim et al., 2017). Besides that, in terms of education, majority of participants are from low education levels. Although the studies are of Asian culture, there was no indication that specific ethnic groups were reported in the reviewed manuscripts.

Although culture might play a role, there are limited information regarding this, and this might limit the interpretation on the role of culture in explaining the relationship between debts and mental health. In general, it might be difficult to attribute the findings to a specific ethnicity. Although there is an intention to explore the cultural explanations on the relationship between debts and loans, this is limited due to the limited cultural information provided in these reviewed manuscripts. From the current manuscripts, none specifically address how their specific culture encourages or discourages debt and explains the relationship between mental health and debt. This is important as indebtedness and the type of debt are found to be related to individuals who hold the value that money leads to prestige and power (Henchoz et al., 2019), or in other words, materialism.

It is argued that materialistic values in some Asian cultures hold more strongly compared to the West. For example, one study finds Singaporean women put greater emphasis on partner status and greater materialism-related happiness compared to American women (Li et al., 2015). However, a study by Awanis et al. (2017) with participants from China, Thailand, India, and the United States extends this understanding of materialism in Asia as a collective-oriented materialism, a belief system that places value on possessions for how they symbolize and signal the capability to grant oneself and others status, how they help the individual comply with social expectations, and how it shows belongingness to a preferred reference group and fulfills their perceived social responsibilities. It is shown that participants in Asia hold this collective-oriented materialism more highly than those in the United States. Hence, it can be argued that the risk of debt in Asia is driven by the pressure of having possessions to show one belongs in that society. Combined with the negative perception some Asian countries have about mental illnesses (Venkatesh et al., 2015; Pang et al., 2017; Huang et al., 2019), this hypothetically may further discourage individuals facing mental health problems with their debt to seek assistance in time, which requires further extensive research. Hence, more research is needed to explore the influence of culture toward debt and the psychological consequences of debt.

Third, in terms of methodology, the use of quantitative methods in these studies incurs several strengths and limitations. Quantitative research is defined by how things are measured or counted, the distribution of the subject matter, how large an object is, how many of the thing is available, and how likely it is to meet the object that is discussed (Lune and Berg, 2017). Quantitative research is argued to be more reliable than qualitative research as it aims to eliminate external variables (Carr, 1994). From the understanding that debt is a widespread phenomenon, the quantitative method provides access to a large sample as a majority of the found manuscripts use a large number of participants. However, one of the limitations of confining to only quantitative methods is that it leaves out the meaning and explanations as to the relationship between debt and mental illness. For example, through interviews with self-harm victims, it is found that debt elicits feelings of fear of repayment and fears of benefit changes increased despair and self-harm (Barnes et al., 2016). Among student parents who reported feeling anxious or depressed, it is understood that these conditions stem from feelings of not being able to contribute properly as a parent to manage their debt (Gerrard and Roberts, 2006). Therefore, both qualitative and quantitative data are required to obtain a more holistic view of the phenomenon of debt and mental illness. More studies in the future may consider the application of mixed methods to achieve this goal.

Fourth, it is observed that few manuscripts report types of debts. Sweet et al. (2013) also noted that different types of debt may have different psychological, social, and material meanings and may occur in different contexts. Hence, their call to action to investigate the different types of debts and the context with which these debts occur. Besides that, few manuscripts report the operational definitions of debts that are used in the individual studies of debt and mental illness. According to Heppner et al. (2007), operational definitions serve as the primary constructs of a study. Issues arise when different operational constructs are formed, which may lead to different results. Defining key concepts becomes more important in research due to the different ways the definitions and concepts are understood due to the background of the readers such as their language, education, and cultural differences (Van Mil and Henman, 2016). Defining operations becomes more critical in the study of debt as there are different types of debt, such as credit card debt, student loans, personal loans from friends of family, and general debt (Hoeve et al., 2014). However, the studies found either do not define debt or use self-reported measures of debt, which poses a risk to the accuracy of the measurement of debt. Currently, there is evidence among participants in England that the type of debt may have little effect on common mental disorders such as depression, anxiety, and obsessive–compulsive disorder, but the number of debts does have an effect (Meltzer et al., 2012). This highlights the need for research articles to be clear in how many types of debts are being explored; this raises the limitations of Yes and No responses as it lacks information to provide a more holistic view of the effects of debt on mental health. In an Asian context, it would be informative to explore if debt types also have specific effects on mental health. Overall, it is recommended to apply standard measures of debt that have been used in other research such as calculating debt-to-income ratio, which can be defined as the ratio of monthly debt payments to monthly pretax income (Kim et al., 2017); the use of debt payment-to-income ratio has been shown to be effective as a measure of borrowing constraints (Johnson and Li, 2010).

In terms of results among the studies that relate debt to depression, the found research supports that individuals in debt experience greater depression (Kaufman, 2015; Mathias et al., 2015; Lee et al., 2016; Shidhaye et al., 2016; Maselko et al., 2018; Seponski et al., 2018). These findings are in line with many findings of previous studies. One study found that being in debt increased symptoms of depression beyond the effects of other socioeconomical factors such as income, wealth, education, occupational status, employment, and earlier mental health (Drentea and Reynolds, 2012). Berger et al. (2015) found that specifically short-term debt was associated with depression among adults in the United States, and this effect is most concentrated among adults age 51–64. Among Finnish participants, debt has a small but significant effect on the anxiety and depression as a 1% increase in debt related to 0.04 increase in probability of having anxiety or depression (French and McKillop, 2017). A class effect is also seen as individuals in low- and middle-income groups, despite having the least amount of debt, experience greater distress over debt; among middle-income groups, the higher the level of debt, the higher the level of depression and anxiety (Hodson et al., 2014).

In addition, only one manuscript found that self-reported anxiety is higher among those in debt (Seponski et al., 2018). Despite the low number of studies in the Asian context, its findings are reflected in other studies. Controlling for generalized anxiety disorder (GAD) symptoms at baseline, and adjusting for sociodemographic factors, exposure to financial stressors after baseline but before follow-up, and history of mood or anxiety disorders, foreclosure increases symptoms of GAD (McLaughlin et al., 2011). UK students not being able to pay bills predicted higher anxiety as explained by financial stress (Richardson et al., 2016). Through interviews, Nissen et al., 2019 reported that students with high debt expressed anxiety about their loans, and despite flourishing at the university, they are terrified about their future with regard to their debt, describing the debt as something that would negatively impact their salaries, house ownership, or ability to repay the debt.

In the study of stress and debt, high levels of stress were significantly related to caregiver's debt burden (Sharma et al., 2018). The population of this study is highly specific; hence, more research is needed regarding how the general population is affected in terms of stress due to debt. The influence of debt on stress has been shown in the general population in previous research. Controlling for self-related health, parental help, and net worth increase in debt amount increases stress in young adults, and it was found that scores on the six-item Kessler Scale increase by 0.08 if total debt is raised by 1000 USD, and credit card debt raises K6 sum of score by 0.22 for every 1,000 USD (Zhang and Kim, 2018). Among college students, it has been reported that increasing student loans had a significant impact on financial stress, and comparing students without debts with those with debts, those who reported having debt within the range of 12,000 to 30,000 USD reported an increase in stress by 0.74 (Britt et al., 2015). Young adults with high debt even after liquidating all assets are associated with higher perceived stress (Sweet et al., 2013).

Two manuscripts found higher suicide ideation among females in debt (Manning et al., 2015; Xu et al., 2015). The risk of suicide ideation among those individuals is supported by previous findings (Turunen and Hiilamo, 2014). In England, having debt increased the odds of suicide ideation alongside being female, belonging in a younger age group, single or widowed, separated or divorced, having problems with alcohol or drugs, and being unemployed or economically inactive (Meltzer et al., 2012). Furthermore, those with several debts compared to those with just one debt were more likely to report suicide ideation; this relationship between debt and suicide ideation is found to be partially mediated by hopelessness.

From manuscripts included in this review, the main findings revolve around establishing a relationship between debt and the mental health issue studied with only one study exploring how income levels affect the relationship between debt and depression (Lee et al., 2016). Hence, more research is needed to explore how certain sociodemographic variables affect the relationship between debt and mental health issues. This would be a topic of interest as it is known that debt experience can differ by age. Besides that, in terms of gender, male and females are found to respond differently toward debt in terms of perceived financial or emotional stress, subjective evaluation of the financial situation, and feelings toward their partners and themselves (Callegari et al., 2019). Even income class affects the experience of debt differently; middle-income Americans are reported to experience high depression and anxiety from having consumer balances, which further increased following the Great Recession, but lower-income consumers saw less of this effect until after the recession, which can be attributed to the lower access and use of credit (Hodson et al., 2014).

Although a conclusion about the state of debt and its effects in Asia cannot be drawn from these few studies, the trend that is observed from these findings is that among these Asian participants, there is evidence that being in debt is positively related to depression, anxiety, stress, and suicide ideation. The study on the effects of debt also needs to be made a primary objective as majority of these manuscripts do not look into debt as a primary factor of mental illness. This calls for research with more precise methodology especially in defining and measuring debt. In addition, other factors that influence the relationship between debt and mental illness need to be explored.

### Limitations

The findings of this study are restricted by several limitations. First, the choice to omit unpublished literature may incur some bias on the findings of this study. The use of highly specific participants studied in these research manuscripts also limits the generalizability of these findings.

## Conclusion

Overall, the present review finds that there is lack of research on the effects of debt on mental health issues such as depression, anxiety, stress, and suicide. Methodologically, there is a need to understand the context behind the relationship between debt and mental health issues and clearer definitions of debt.

## Author Contributions

NA, ET, NI, and NC contributed to conception and design of the study. NA and ET organized the databases and wrote the first draft of the manuscript NA, ET, and NI performed the statistical analysis. MM, AZ, RI, TT, ET, and NA revised the manuscript. All authors contributed to manuscript revision, and read and approved the submitted version.

## Conflict of Interest

The authors declare that the research was conducted in the absence of any commercial or financial relationships that could be construed as a potential conflict of interest.
